# A Low-Cost Humidity Control System to Protect Microscopes in a Tropical Climate

**DOI:** 10.5334/aogh.2585

**Published:** 2020-02-13

**Authors:** Anders J. Asp, Christina M. Webber, Evan N. Nicolai, Gabriel Martínez-Gálvez, Victoria S. Marks, Ephraim I. Ben-Abraham, John W. Wilson, J. Luis Lujan

**Affiliations:** 1 Mayo Clinic Graduate School of Biomedical Sciences, Mayo Clinic, Rochester, Minnesota, US; 2 Department of Infectious Diseases, Mayo Clinic, Rochester, Minnesota, US; 3 Department of Neurologic Surgery, Mayo Clinic, Rochester, Minnesota, US; 4 Department of Physiology and Biomedical Engineering, Mayo Clinic, Rochester, Minnesota, US

## Abstract

**Introduction:**

A clean and functional microscope is necessary for accurate diagnosis of infectious diseases. In tropical climates, high humidity levels and improper storage conditions allow for the accumulation of debris and fungus on the optical components of diagnostic equipment, such as microscopes.

**Objective:**

Our objective was to develop and implement a low-cost, sustainable, easy to manage, low-maintenance, passive humidity control chamber to both reduce debris accumulation and microbial growth onto the optical components of microscopes.

**Methods:**

Constructed from easily-sourced and locally available materials, the cost of each humidity control chamber is approximately $2.35 USD. Relative humidity levels were recorded every 30 minutes over a period of 10 weeks from two chambers deployed at the Belize Vector and Ecology Center and the University of Belize.

**Results:**

The humidity control chamber deployed at the University of Belize maintained internal relative humidity at an average of 35.3% (SD = 4.2%) over 10 weeks, while the average external relative humidity was 86.4% (SD = 12.4%). The humidity control chamber deployed at the Belize Vector and Ecology Center effectively maintained internal relative humidity to an average of 54.5% (SD = 9.4%) over 10 weeks, while the average external relative humidity was 86.9% (SD = 12.9%).

**Conclusions:**

Control of relative humidity is paramount for the sustainability of medical equipment in tropical climates. The humidity control chambers reduced relative humidity to levels that were not conducive for fungal growth while reducing microscope contamination from external sources. This will likely extend the service life of the microscopes while taking advantage of low-cost, locally sourced components.

## Introduction

**Figure 1:**
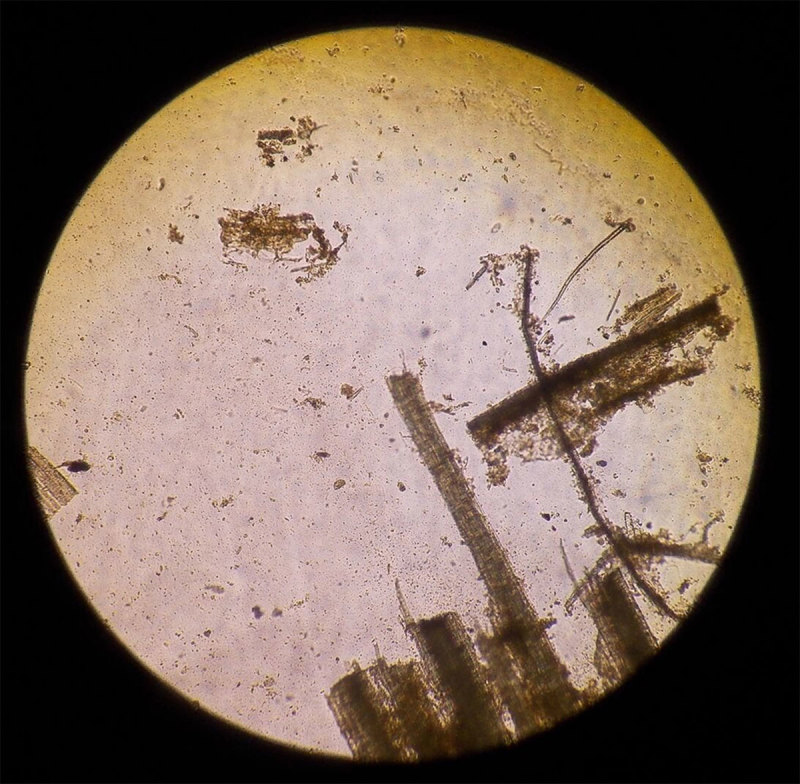
Representative bright field image from a microscope in Belize showing obstructed field of view due to accumulation of debris on objective, eyepieces, and internal components. Debris is likely comprised of fungal growth and dust, containing both organic and inorganic matter.

Infectious diseases are among the most significant contributors to global morbidity and mortality. Despite improvements in clinical care, tuberculosis and malaria were responsible for a combined 2 million deaths worldwide in 2017 [1,2]. Maintaining microscopy equipment is paramount in clinical settings, particularly in tropical climates or underserved regions of the world where microscopy is the gold standard for diagnosis of endemic infectious diseases such as malaria, tuberculosis, and coccidiosis [3]. In these regions, optical components of microscopes can accumulate fungal growth or debris (Figure 1) due to high relative humidity levels, improper storage, or poor maintenance [4,5]. These conditions can 1) impair the accurate identification of microorganisms responsible for infectious diseases, 2) shorten the service life of diagnostic equipment, and 3) result in added costs. In fact, multiple microscope manufacturers explicitly state in their usage and warranty policies that microscopes should be stored in environmental conditions free from debris and with low relative humidity levels [6,7,8].

Microscopy-based organism identification remains vital for the accurate and time-efficient diagnosis of many infectious diseases. The high relative humidity in warmer tropical regions, often exceeding 85%, increases the likelihood of fungal growth on microscope optics. Controlled studies have demonstrated that fungi grow in relative humidity levels of 80%, while growth is not observed at levels below 50% [4]. This finding is consistent with other recommendations that relative humidity levels be kept below 65% to mitigate fungal growth on optical components [5]. Fungal growth resulting from poor laboratory equipment maintenance has already been shown to impair the microscopy-supported diagnosis of tuberculosis in tropical environments like Tanzania and Ethiopia [9,10,11]. While vendor-supplied plastic microscope covers are available, they can easily become soiled or ripped, making them ineffective at reducing debris accumulation and growth of biological contaminants on microscope surfaces. The replacement of dysfunctional equipment through suboptimal maintenance is cost-prohibitive and preventable through changes in standard operating procedures. For example, a new Olympus optical microscope (Olympus Corporation, Center Valley, PA) can range from $1,400–$2,000 USD at the time of this publication. Many medical laboratories, particularly in Central America and Africa, do not have the resources for repeated replacement of diagnostic equipment.

There is a substantial need for a strategy to improve the functional operating longevity of microscopy equipment within tropical regions and underserved regions of the world where proper maintenance is particularly challenging. To this end, the Mayo Clinic Initiative for Medical Equipment Sustainability (IMES) has partnered with the Ministry of Health in Belize and the University of Belize to develop and implement a low-cost, sustainable, easy to manage, low-maintenance, passive humidity control chamber. The objective was to both reduce debris accumulation and microbial growth on the optical components of microscopes. Herein, we describe the criteria for use, material list, and fabrication instructions. We also characterize chamber performance with the goal of facilitating medical equipment sustainability.

## Materials and Methods

**Figure 2:**
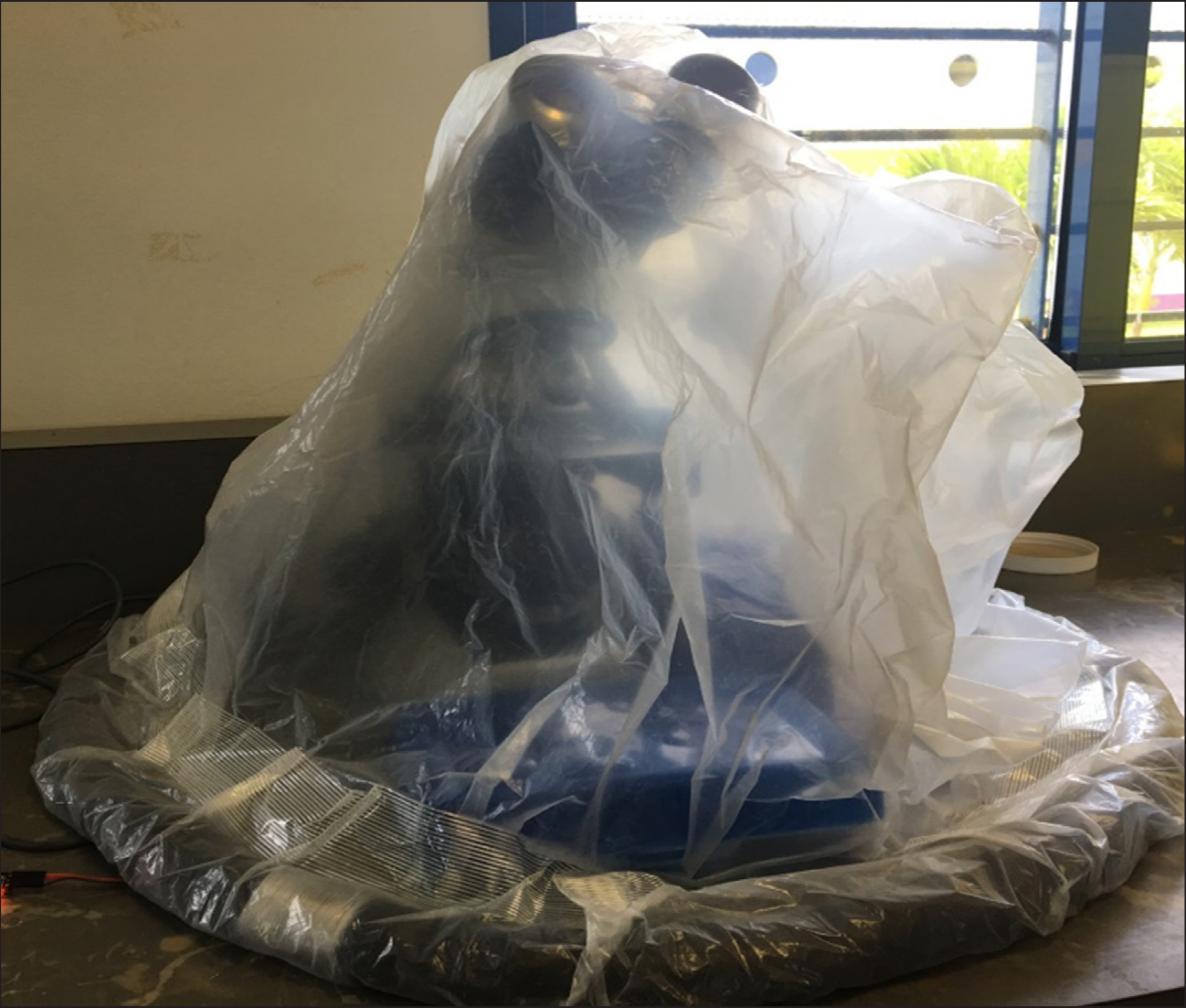
Humidity control system containing microscope.

A passive humidity control chamber (Figure 2) was designed to prevent accumulation of debris and microbial growth onto microscopes or similar sensitive medical diagnostic equipment. This humidity control chamber was optimized for use in environments where there is observed:

- Relative humidity consistently above 65%
- Accumulation of debris (dirt, dust, etc.) on equipment surfaces
- Fungal growth on the surface of the optical components of microscopes and other medical equipment that is sensitive to moisture such as otoscopes and endoscopes
- Improper storage conditions that do not comply with manufacturer recommendations

**Figure 3:**
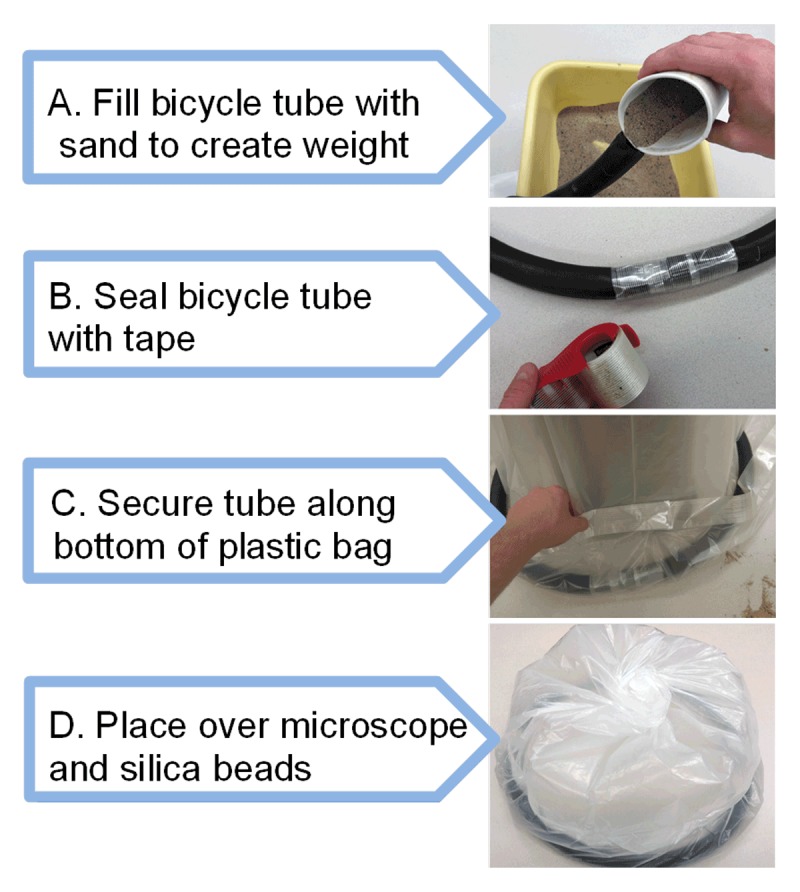
Humidity control chamber fabrication instructions.

The main components of the humidity control chamber consisted of a plastic bag to cover the equipment (Figure 3), a weighted skirt to hold the cover in place and prevent air leaks, and silica beads to absorb water from the air. Weighted skirts for the humidity control chamber were created by sealing a sand-filled, 26-inch bicycle tube with Scotch brand packing tape (3M, St. Paul, MN). Weighted skirts were then placed around the rim of a standard 55-gallon plastic bag and secured with additional packing tape. The finished humidity control chamber was then placed over a microscope and a glass jar filled with 500 mL of reusable silica beads (Figure 2). The cost for building the humidity control chamber, including silica beads, is approximately $2.35 USD (Table 1).

**Table 1:** Material list for humidity control chamber. Similar locally sourced materials may be used as substitutes.

Materials	Cost

500 mL reusable silica beads	~$2.00
Garbage bag (~50 gallon)	~$0.10
500 g sand, packing tape, mason jar	<$0.25
Punctured 26” bicycle tube	$0.00
Glass jar for silica beads	$0.00

Two humidity control chambers were deployed to separate locations in Belize; one in the University of Belize in Belmopan and the other in the Belize Vector and Ecology Center in Orange Walk. Belize was chosen as the initial test location due to consistently high relative humidity levels of approximately 85% and a warm climate. Internal and external chamber relative humidity levels were recorded every 30 minutes using DHT11 humidity/temperature sensors (Adafruit Industries, New York, NY). An Arduino UNO (Arduino, Ivrea, Italy) collected this humidity data over a 10-week period. Relative humidity data was saved to an onboard micro SD card and transferred to the IMES team via email at the end of the 10-week period. Microscopes were left undisturbed for the duration of the study. A time series autoregressive statistical model (JMP, SAS Institute, Cary, NC) was used to determine if significant differences (p < 0.05) existed between internal and external relative humidity values.

## Results

**Figure 4:**
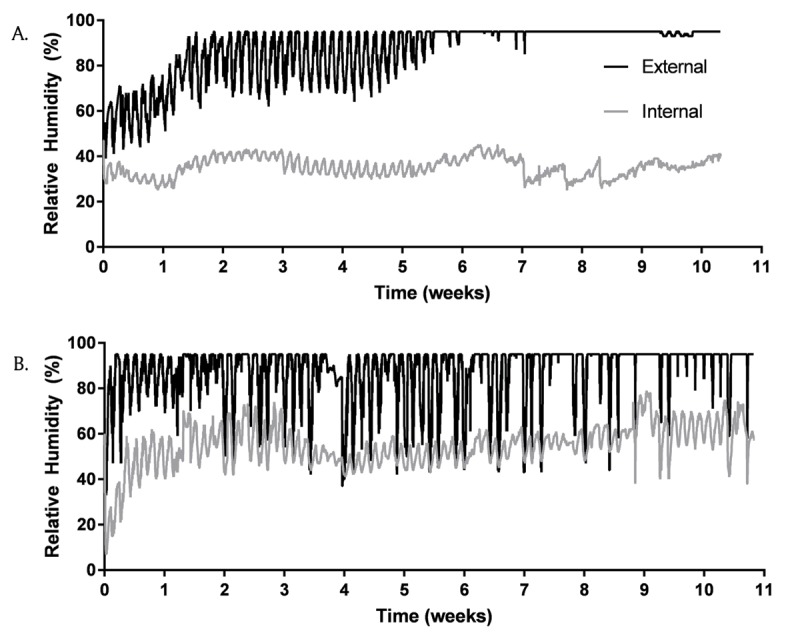
Relative humidity in and out of chamber located at the University of Belize **(A)** and the Belize Vector and Ecology Center **(B)**. Data collected every 30 minutes with DHT11 sensor with an Arduino UNO. An autoregressive model demonstrates external and internal relative humidity are different; p < 0.001.

Data were collected from June 6, 2018 to September 10, 2018. The humidity control chamber deployed at the University of Belize maintained internal relative humidity at an average of 35.3% (SD = 4.2%) over 10 weeks, while the average external relative humidity was 86.4% (SD = 12.4%) (Figure 4A). The humidity control chamber deployed at the Belize Vector and Ecology Center effectively maintained internal relative humidity to an average of 54.5% (SD = 9.4%) over 10 weeks, while the average external relative humidity was 86.9% (SD = 12.9%) (Figure 4B). A time series autoregressive model was independently applied to data from both locations and indicated a significant difference between internal and external relative humidity levels (p < 0.001).

## Discussion

The humidity control chambers effectively maintained an average internal relative humidity level below the recommended limit of 65% [5,12,13]. The performance of the humidity control chambers in tropical climates therefore offers the potential to prevent mold growth. Daily variation in relative humidity was observed in both locations, with large changes prominent at the Belize Vector and Ecology Center location (Figure 4B), perhaps due to the intermittent use of an air conditioning system and/or power outages. The humidity control chamber buffered these variations in relative humidity.

The design of the humidity control chamber is intentionally simple. It is made with components commonly available in most parts of the world that can be substituted with other locally accessible materials. The environmental barrier can be created from any plastic bag large enough to completely cover the microscope. Additionally, the weighted skirt can be constructed from any dry, heavy material (e.g., pebbles, beans, etc.) that keeps the plastic barrier flush with the table surface. It is recommended that the weighting material be contained using a bicycle tire tube. A punctured bicycle tire may be acquired at no cost from local repair shops. Rice or a similar desiccant could be used to dehumidify the internal environment in the absence of silica beads. Uncovered glass jars were used to hold the silica beads; however, a perforated lid, muslin bag, or similar vessel may be used instead to avoid spilling. Verification of new materials would be required to ensure equivalent functionality. Although our study ended before the silica beads lost efficacy, our data indicates that 500 g or reusable silica bead desiccant will control humidity levels around an undisturbed microscope for at least 10 weeks before recharging the desiccant becomes necessary. When the humidity control chamber is deployed around heavily used medical equipment, the service life of the beads may be reduced. We recommend the use of blue indicating silica beads which change color as they absorb moisture to notify laboratory personnel they must be recharged. An analog or digital hygrometer may also be deployed within the chamber to ensure relative humidity is kept below 65%.

This is an initial effort to maintain humidity levels in a low-cost, low-maintenance storage device for microscopes in high humidity climates. While microscopes were initially selected for their sensitivity to high relative humidity, it is important to note that our humidity control chamber can be used to protect any small moisture-sensitive equipment; including basic research equipment, medical electronics, chemicals, or drugs. Preliminary results assessing humidity reduction potential are promising; however, continued longitudinal relative humidity data acquisition is necessary to fully characterize the humidity control chamber. Additional steps, such as assessment of fungal growth and demonstration of improved sustainability of microscopy equipment are needed. Field studies with additional humidity control chambers under typical usage conditions will provide information to further characterize the impact of the system.

## Conclusion

Sustainability of medical and diagnostic equipment is critical in clinical settings in tropical climates or underserved regions of the world. The low-cost humidity control chamber described herein reduced the relative humidity within the chamber to levels that were not conducive for fungal growth in the tropical climate of Belize. The physical barrier in this system also offered protection from debris. Together, these can extend the usable lifespan of valuable diagnostic equipment and reduce the need for allocating additional resources for repeated replacement of diagnostic equipment.
